# High Power Q-Switched Thulium Doped Fibre Laser using Carbon Nanotube Polymer Composite Saturable Absorber

**DOI:** 10.1038/srep24220

**Published:** 2016-04-11

**Authors:** Maria Chernysheva, Chengbo Mou, Raz Arif, Mohammed AlAraimi, Mark Rümmeli, Sergei Turitsyn, Aleksey Rozhin

**Affiliations:** 1 Aston Institute of Photonic Technologies (AIPT), Aston University, Aston Triangle, Birmingham, B4 7ET, United Kingdom; 2 The Key Laboratory of Specialty Fiber Optics and Optical Access Network, Shanghai University, 200072, Shanghai, China; 3 Physics Department, Faculty of Science, University of Sulaimani, Sulaimani, Iraq-Kurdistan Region; 4 Engineering Department, Al Musanna College of Technology, 314, Muladdah Musanna, Sultanate of Oman; 5 Leibniz Institute of Solid State and Materials Research, IFW, Helmholtzstraβe 20, 01069 Dresden, Germany; 6 Novosibirsk State University, 2 Pirogova Str., Novosibirsk, 630090, Russia

## Abstract

We have proposed and demonstrated a Q-switched Thulium doped fibre laser (TDFL) with a ‘Yin-Yang’ all-fibre cavity scheme based on a combination of nonlinear optical loop mirror (NOLM) and nonlinear amplified loop mirror (NALM). Unidirectional lasing operation has been achieved without any intracavity isolator. By using a carbon nanotube polymer composite based saturable absorber (SA), we demonstrated the laser output power of ~197 mW and pulse energy of 1.7 μJ. To the best of our knowledge, this is the highest output power from a nanotube polymer composite SA based Q-switched Thulium doped fibre laser.

In recent years mid infrared (mid-IR) light sources around 2 μm have attracted a great deal of attention^1^,^2^. Such sources are in demand for various applications, including: atmospheric gas analysis^3^,^4^,^5^, biomedical diagnostics^6^,^7^, remote sensing^8^, plastic welding^9^ and THz generation^10^. Additionally, optical free-space communication operating in the 2–2.5 μm spectral window has been recently examined^11^,^12^. High pulse energy and high peak power light sources, operating in mid-IR, are highly desirable for light detection as well as ranging applications^8^,^13^. The Holmium or Thulium doped, and quantum cascaded based solid state lasers have been developed over the past few decades. However, it is regarded that a promising solution might be found with the optical fibre based laser systems. Such systems possess numerous attractive characteristics: they are alignment free, compact, featuring excellent heat dissipation, and low maintenance cost etc. Thulium-doped fibre (TDF) with broad gain bandwidth offers opportunities for wide tuneability (from 1800 to 2100 nm) and short pulse duration generation. These benefits make TDF a captivating platform for the light sources in this wavelength region^1^. Q-switched TDF lasers are already actively used in the scientific and industrial applications, such as, LIDAR and ranging^13^, supercontinuum generation^14^,^15^, and nonlinear frequency conversion for mid-IR^16^,^17^. Active Q-switching technique using external controller can provide for variability of the laser performance including pulse duration and repetition rate^18^. However, bulky components, such as, modulator and preferential complex electronics would be the significant barrier towards portable and robust systems design. Passive Q-switching utilising fibre nonlinearity or physical saturable absorber (SA) based intensity modulator, is a more attractive technique due to its less complexity, versatile design and low cost. Fibre nonlinearity based effective SAs including NOLM^19^, NALM^20^,^21^ and the nonlinear polarisation rotation (NPR)^22^ have many attractive features and are well known and used in the field of ultrafast lasers. However, such nonlinearity SA based laser systems are, typically, less immune to environmental perturbations.

A material based SA uses a medium that shows transparency when the incident light is of high intensity while behaves as a loss element when the launched radiation is weak. Such components are easy to use and, importantly, they provide the system with the self-starting operation. To date, various types of SA has been proposed and demonstrated for fibre laser Q-switching including carbon nanotubes (CNT)^23^,^24^,^25^,^26^, graphene/graphene oxide^27^,^28^,^29^,^30^,^31^,^32^,^33^, molybdenum disulphide (MoS_2_)^34^,^35^,^36^, tungsten disulphide (WS_2_)^37^,^38^, topological insulator^39^,^40^,^41^,^42^, gold nanoparticle^43^, and semiconductor saturable absorber mirrors (SESAM)^44^. Although SESAM technology used to be a popular choice in the pulsed laser market owing to its controllable saturable absorption property and versatile design, the apparent drawbacks are arguably high manufacturing cost, bandwidth limitation and operation lifetime. Furthermore, SESAM is not particular fibre-friendly which means they are not very easy to incorporate into a fibre laser resonator. In addition, at 2 μm, SESAM technology up to now has only been limited to few suppliers^44^. Recent developed materials such as 2D materials (MoS_2_, WS_2_) and topological insulator or gold nanoparticles have good potential due to the broadband response. Nevertheless, high power operation of Q-switched 2 μm TDFL with such SAs have not been demonstrated yet. Carbon materials, such as graphene and carbon nanotubes have been proven already as efficient SA for TDF laser Q-switching in both fibre and bulk format^23^,^24^,^25^,^27^,^28^,^29^,^30^,^31^,^45^. However, in this case it is very challenging to achieve high output power keeping the all-fibre property^31^ and using only standard fibre components^23^,^29^.

CNTs with various spatial scales, chirality and related geometry, featuring specific optical absorption have been investigated intensively as a novel type of SA for implementing pulsed fibre laser systems in the past decade^46^,^47^,^48^,^49^,^50^,^51^. Apart from the bundle engineering for efficient photonic application, CNT can be effectively embedded into polymer composite^52^,^53^. The format of polymer composite film based SA offers an elegant route to the all-fibre configuration with a low manufacture cost^54^. However, such CNT polymer composites are difficult to operate in the high power regimes at near infrared region (1.0-1.5 μm) due to the intrinsic thermal degradation of the polymer. Moreover, complicated procedure is necessary to fulfil the requirement of embedding CNT into polymer material with high thermal endurance property^55^.

In this report, we demonstrate a TDF laser passively Q-switched by CNT polymer composite SA with high output power. We use polyvinyl alcohol (PVA) as the polymer matrix to host the CNT. The laser configuration also features a double feedback “Yin-Yang” structure whereas the unidirectional oscillation of the laser has been implemented through the mutual feedback operation of the NOLM/NALM hybrid configuration. Such approach helps to ease the configuration of the laser resonator by removing in-line fibre optical isolator simplifying cost, manufacturing and reducing corresponding insertion losses. Such a cavity configuration opens opportunities to predefine the operation direction as well as output power distribution of the laser through the design. Evidently, the double clad TDF with free space bulky may provide much higher output power. However, this solution lacks the merits of the all-fibre concept^31^. With the help of CNT SA, the genuine implementation of an all-fibre TDF laser can be achieved. With the maximum output of 197 mW at 1.9 μm, the proposed laser, to the best of knowledge, demonstrates the highest output power from a CNT polymer based Q-switched TDF laser. This laser is attractive for a range of applications, such as nonlinear frequency conversion, frequency comb generation, and metrology and many others.

## Results

**Figure 1:**
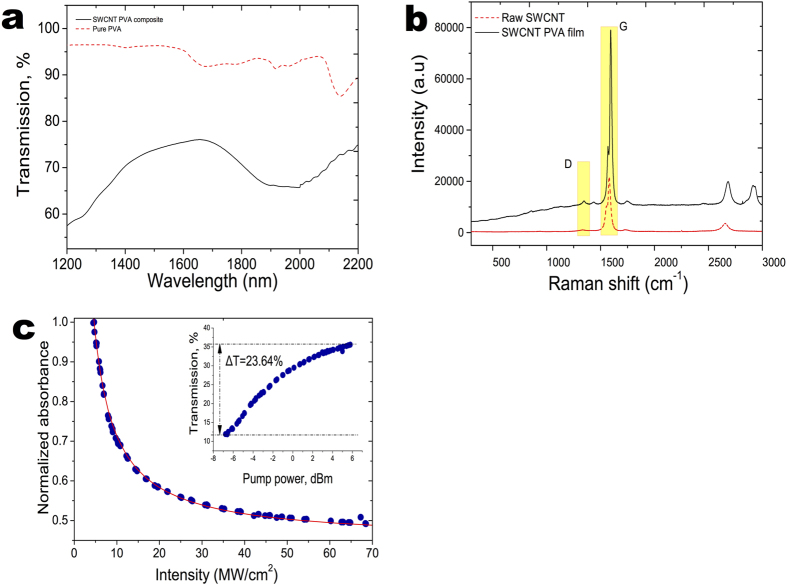
Optical characterisation of SWCNT PVA SA. (**a**) linear optical absorption measurement of SWCNT PVA film and pure PVA. (**b**) Raman spectrum measurement of the SWCNT PVA film and raw SWNT. (**c**) Nonlinear optical absorption measurement of the SWCNT PVA SA.

We fabricated a SWCNT polyvinyl alcohol (PVA) composite with broad absorption band (~300 nm) with the band maximum at 1950 nm (described in Methods and shown in Fig. 1a). This has been obtained using laser ablation SWCNTs with mean diameter of 1.55 nm (+/−0.2 nm). A measured Raman spectrum has been shown for the initial SWCNT powder and SWCNTs-PVA film (Fig. 1b). Both spectra show strong G bands at 1594 cm^−1^ and an insignificant increase in the D- band at 1345.8 cm^−1^ for the composite sample. Thus, the composite preparation procedure resulted in introducing of minor defects in SWCNTs, however, does not affect electronic properties of initial SWCNTs. The reason we use PVA is its proved sophisticated embedding efficiency for hosting SWCNT. As shown in Fig. 1a, pure PVA film possesses low absorption at the 2 μm wavelength region^15^. Additionally, the saturable absorption study by Z-scan method reveals the excellent stability of SWCNT-PVA composite at 1800 nm spectral range due to reduced OH group when compare with composites at 1500 nm spectral range^56^. A typical measured nonlinear optical absorption of the SWCNTs PVA composite is shown in Fig. 1c. At the particular 2 μm windows, the measured non-saturable absorption is α_ns_ = 60% increasing to 80.5% with the minimum absorption modulation depth of α_0_ = 19.5%, corresponding to the film transmission modulation depth of 8%. The relatively large non-saturable loss may attribute to the non-resonance tubes, amorphous carbon, metal catalyst, insertion losses in FC/PC connector due to thickness of SWCNT PVA film and minor scatters from tubes at this particular wavelength region. Further optimisation of tube selection with optimal polymer matrix may result in lower non-saturable losses in the proposed SA. The measured absorption saturation intensity is 1.22 MW/cm^2^. Such values are typical for polymer-based composite films with dispersed SWCNTs^54^. The pronounced high modulation depth guarantees that the resultant SA film can effectively initiate pulse regime operation. The fabricated sample exhibits high thermal damage threshold. In^63^ we have demonstrated that the PVA-based sample with SWCNT can endure an optical fluence of at least 3.46 mJ/cm^2^ at ~2 μm wavelength band without any significant damage.

**Figure 2:**
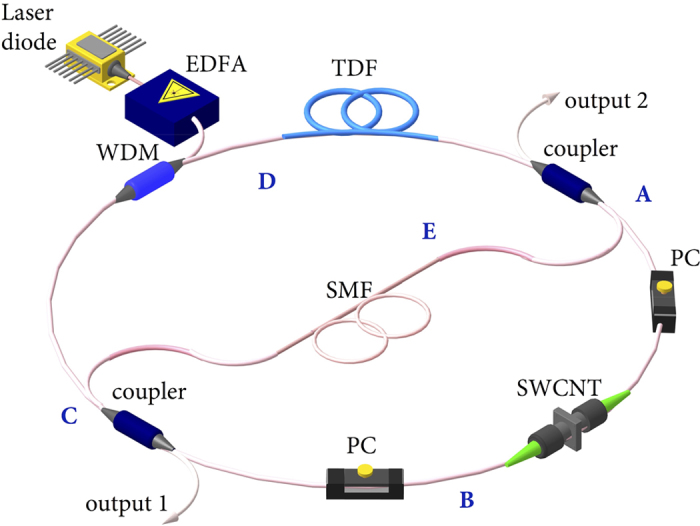
Schematic picture of the NOLM-NALM SWCNT hybrid Q-switched TDFL. A fibre laser built in Yin-Yang cavity configuration including high concentration thulium-doped fibre (TDF), polarisation controllers (PCs), 1550/2000 wavelength division multiplexor (WDM), SWCNT dispersed in PVA-based film sandwiched between two optical connectors, two output couplers with variable coupling ratios, and 1550 nm Fabry-Perot laser diode amplified by EDFA to the maximum pump power of 1.2 W. The total lengths of NOLM (ABCEA), NALM (CDAEC), and common part are 3.5, 3.8, and 0.7 m correspondingly.

The schematic of the Q-switched Tm-doped fibre laser is shown in Fig. 2. The cavity consists of two coupled nonlinear fibre loop mirrors: NOLM (ABCEA) and NALM (CDAEC) and an intersection (AEC). The total lengths of the sections are 3.5, 3.8 and ~0.7 m, correspondingly. Under this hybrid scheme, one fibre loop mirror acts as a feedback to the other. In the experiment, a 1 m Tm-doped single-mode single clad fibre (produced in the FORC, Moscow) with a nominal absorption of 60 dB/m at 1550 nm was used as the active medium. The Tm-doped fibre was pumped via wavelength division multiplexor (WDM) by a 1550 nm, 80 mW semiconductor Fabry-Perot laser diode, amplified through a commercial Er-doped fibre amplifier (IPG Photonics) up to a maximum power of 1.2 W. The SWCNT SA sandwiched between two optical fibre ferrules are placed in NOLM part of the laser. Two in-line polarisation controllers are placed prior to the inputs of the SWCNT module in both directions to ensure proper cavity birefringence. The appropriate adjustment of the polarisation controllers provides a stable Q-switching operation and optimise pulse regime at higher powers. To confirm that the Q-switching operation here was induced by SWCNT polymer film, we deliberately remove the SA from the laser cavity. No Q-switching regime could be found withing the current laser geometry consequently with the available pump and manipulation of the polarisation controller.

**Figure 3:**
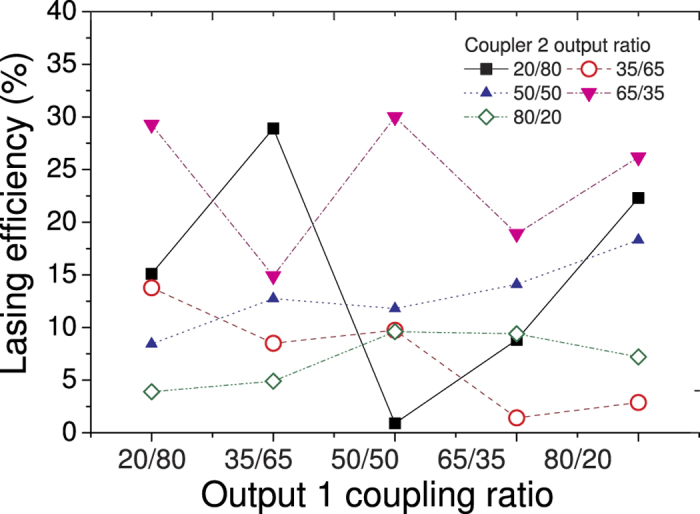
Laser output dynamics against the coupler ratios.

**Figure 4:**
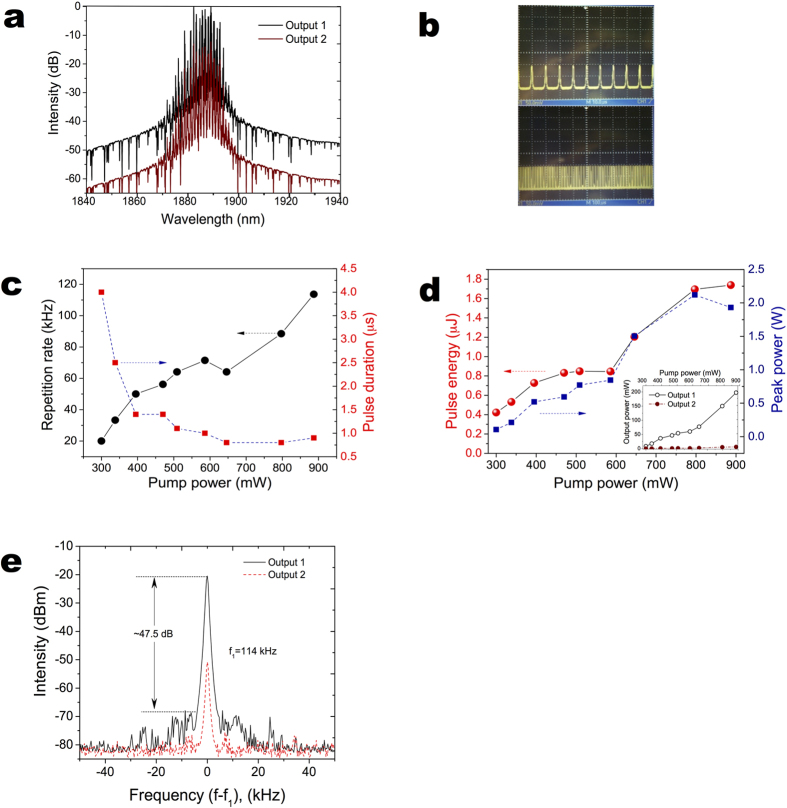
Output pulse properties at optimised condition. (**a**) optical spectrum, measured at different laser outputs. (**b**) recorded oscillograms from output 1 at different sampling times (i) 10 μs; (ii) 100 μs; (**c**) repetition rate and pulse duration variation from output 1 against pump power; (**d**) output pulse energy and peak power variation from output 1 against pump power. Inset: laser efficiency from different output ports; (**e**) measured RF spectra at different output port.

In our experiments, we used a number of couplers with the coupling ratio of 20/80 (the number before ‘/’ means output coupling ratio, the number after ‘/’ means for laser cavity coupling ratio), 35/65, 50/50, 65/35, and 80/20 respectively. The laser efficiency of output1 against various coupling ratios at output2 has been plotted in Fig. 3a. From Fig. 3a, we can infer that when the output1 and output2 coupling ratio is 50/50 and 65/35 individually, the laser operates at the optimum status. The laser has a slope efficiency of 30% with output power of 197 mW. It is worth noting that the output radiation is free from the residual pump at 1550 nm, proving full absorption of pump light in the active Tm-doped fibre. With the optimised coupling ratio, the laser threshold is about 200 mW and stable Q-switching operation could be obtained at up to ~1 W pump power. The maximum output power is 197 mW, which is the highest among all SWCNT Q-switched fibre lasers with polymer composite based SA sandwiched between two fibre ferrules. The pulse width is measured to be 0.9 μs, which is a typical value for SWCNT Q-switched laser pulses^24^. The laser spectrum from Output1 in both logarithmic and linear scale is shown in Fig. 4a. The spectrum is centred at 1885 nm with an envelope bandwidth of about 6 nm at −3 dB level. A number of peaks appear in the spectrum, showing that many longitudinal modes oscillated simultaneously because of high cavity gain and laser peak power without mode selecting elements in the cavity^31^. Due to the low nonlinearity in this particular wavelength region, the fibre nonlinear birefringence induced filter effect does not provide enough bandwidth for mode selection. By tuning the intracavity PC, we could achieve wavelength selection within a limited range. However, the spikes still exist. We anticipate that further incorporating high nonlinear or high birefringence element could improve the mode structure in the optical spectrum. The laser pulse trains obtained at the higher output power levels of 197 mW are shown in Fig. 4b. As it can be seen, the output laser pulses are stable. No noticeable intensity fluctuation can be observed. The pulse repetition rate increases relatively linearly from 20 kHz at threshold pump to 114 kHz at the maximum power (Fig. 4c). Conversely, the pulse width decreases nonlinearly from 4 to 0.9 μs. This corresponds to typical pulse behaviour of Q-switched lasers. The evolution of the pulse energy and peak power against pump power is shown in Fig. 4d. The peak power increases with pump up to the maximum peak power of 1.9 W while the pulse energy shows an increase with the maximum pulse energy of 1.7 μJ. We measured the RF spectra of both output ports as shown in Fig. 4e. Output1 shows a signal to noise ratio (SNR) of 47.5 dB when the output power is 197 mW. While, at the same time, output2 shows a 20 dB less SNR due to lower output power. The demonstrated laser operated stable in the lab condition for one day without any noticeable degradation of performance.

## Discussion & Conclusion

The operation mechanism of the proposed fibre laser can be explained as following. Owing to the low photon energy at 2 μm region, laser pulsation based on nonlinearity would generally require long fibre cavity length. A short cavity is not able to generate laser pulses. SWCNT SA was therefore used to initiate the optical pulse circulating in the laser cavity. The laser can then be regarded as a result of hybrid feedback scheme based on either NALM or NOLM. It should be stressed that the initial optical power distribution propagating in clockwise and counter-clockwise direction is defined by the ratio of main coupler in both mirrors. Considering NALM, the optical pulses with higher power going through the clockwise direction (CDAEC, c. direction) would acquire more phase shift compared to the counter clockwise pulse propagation (CEADC, c.c. direction) because pulses would be amplified by the active gain from the beginning of the propagation^21^. Under this scheme, coupler 2 functions as only a passive loss element within the NALM, and coupler 1 serves as the NALM main coupler as well as the laser output. On the other hand, for NOLM operation scheme, laser pulses propagating in the c. direction (ABCEA) can gain less phase shift compared to the light propagation in the c.c direction (AECBA) due to the extra loss experienced by the light when travelling through the SA element^19^. At this scenario, coupler 1 functions as the passive loss element, and coupler 2 works as the NOLM main coupler and the laser output. Nevertheless, at certain coupler ratios, pulses propagating in the c.c. direction are able to acquire more nonlinear phase shift than the c. direction in both loop mirrors due to the interplay between the main coupler ratio and the extra loss element within each loop mirror. After propagating through the mirrors, light pulses come back to the primary fibre couplers with the same power distribution as in the beginning. The distinction between the accumulated nonlinear phase shifts from the counter propagating pulses determines the interference pattern at the main coupler and therefore transmission loop mirror^57^,^58^. Therefore, the high intensive pulse is transmitted to the output while low intensive radiation is reflected back to the mirror. The similar happens with the pulse temporal profile. The high power peak region and the low intensity wings region of the pulse acquire different nonlinear phase shift during their propagation through the loops. As a result, the interference at the main coupler provides intensity dependent transmission, which is analogous to pulse formation mechanism of conventional saturable absorbers. It is this interference pattern that defines the pulse shaping and transmission properties of the nonlinear loop mirrors such that implementing single direction oscillation without the necessity of an isolator. If transmission of the NALM is higher than NOLM, output 1 will be the primary output of the laser and vice versa. When both mirrors have similar transmission, the laser can have synchronised outputs from both couplers.

**Figure 5:**
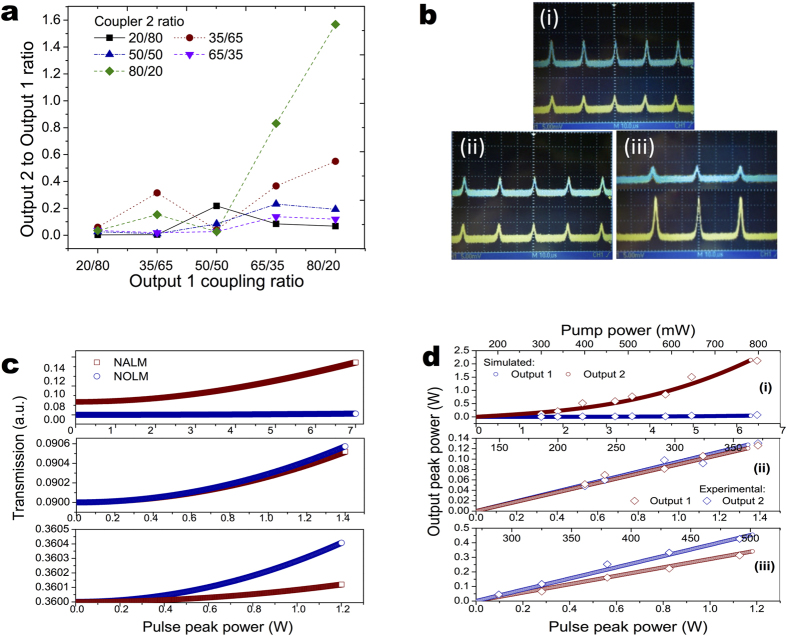
(**a**) Output power ratio of output 2 to output 1 at various coupling ratio. (**b**) Typical oscilloscope traces of laser output with different ouput1 (lower yellow trace) to output2 (upper blue trace) coupling ratios, (**c**) NOLM and NALM transmission and (**d**) output peak power at variable pulse peak power at 35/65 to 65/35 (i), 65/35 to 80/20 (ii), 80/20 to 80/20 (iii) coupling ratios, correspondingly.

Despite the fact that the laser operates in single direction, the laser also features two outputs from which individual output powers can be defined by the two couplers. Varying the coupling ratios directly alters the total power distribution within the cavity, and hence modifying its gain and pulse dynamics behaviour. It shows that under the Q-switched operation the laser cavity is directionally asymmetric. There is a Q-switch priority direction depending on output coupling ratios. The ratio of the power at output 2 against output1 under various coupling ratio at the maximum pump power is illustrated in Fig. 5a. As it can be seen in Fig. 5a, we are able to obtain the output power switching between the output 1 and output 2 by tuning the coupling ratios while maintaining unidirectional lasing without isolators. This switching capability has been further proved when observed using a two channel oscilloscope as shown in Fig. 5b. Such switching behaviour is mainly due to the interchange of reflectivity (transmission) between the NOLM and NALM. The temporal transmission of short pulses in NOLM strongly depends on coupler ratio ρ, pulse peak power P(t), nonlinearity γ and length L of the fibre loop according to equation (1). Whereas the transmission of short pulses in NALM is also determined by the total active fibre gain G based on equation (2) 58:


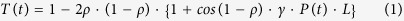



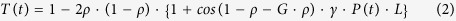


The laser configuration and lengths of the cavity fibres during experiments was, therefore, strictly preserved unchanged to guarantee that the NOLM and NALM reflectivity variation was induced only by alternated coupler ratios. The two laser outputs have similar optical spectrum (Fig. 4a), as well as almost identical lasing thresholds (inset in Fig. 4d).

Figure 5c presents the theoretical calculation of the NOLM and NALM transmission against the intracavity pulse peak power, using Eqs (1) and (2) for three sets of output 1 and 2 couplers ratio, correspondingly: (i) 35/65 to 65/35 (ii), 65/35 to 80/20 (iii), 80/20 to 80/20. Because the asymmetry of the NOLM and NALM has been enhanced by the passive couplers, their loss factors were taken into account when performing the calculation. As it can be seen, the transmission of both NOLM and NALM increases monotonically with elevating intracavity pulse peak power. By varying the ratio of the main coupler of each loop mirror, the transmission of NOLM could evolve faster, slower and similar to that of the NALM, therefore exhibiting the output switching feature. Using the theoretical calculation, we could estimate the increase of the output pulse peak power at both outputs against pulse peak power in the cavity (Fig. 5d). Figure 5d shows that the variation of the simulated output pulse peak power is in a very good agreement with the increase of the measured output pulse peak power along pump power levitation. This, again, proves that in the case when the NALM transmission is growing faster than NOLM, the laser power at output 1 dominates over output 2 and vice versa (Fig. 5b).

It should be noted that the average output power could be further increased if only it weren’t limited by the maximum power of pumping source. As it is seen from inset in Fig. 4d, the output power saturation wasn’t observed during the pump power increase. The laser can hence generate stable pulses without component degradation and additional alignment during high-power operation. A further step to improve the environmental stability can be the utilisation of the polarisation maintaining components. Moreover, nonlinear effects, which are detrimental for conventional Yb- and Er-doped fibre lasers under high power regime operating at wavelength bands of 1 and 1.5 μm, correspondingly, have a higher threshold at longer wavelength of 2 μm^59^,^60^. This allows further power scaling without significant pulse deterioration. However, for radical power scaling, other techniques of the SWCNT SA implementation should be used, which target to increase mode field diameter of the laser beam launched on the SA^23^,^29^. Also, other polymers composites^61^ or free-standing SWCNT films^62^ can be used to achieve higher thermal stability of SA.

The proposed and demonstrated fibre laser system represents a crucial step in realising a stable, versatile, fully monolithic and high–performance laser source operating at pulse regime. The switching mechanism between laser outputs with fully synchronized pulse trains allows the user to use a single laser source for two different types of measurement or imaging techniques such as confocal imaging and total internal reflection fluorescence microscopy, pump-probe and fibre optical telecommunications in photonic flip-flop memories, optical loop buffers, secure telecommunications, pulse sampling devices .

In conclusion, we have proposed and demonstrated a low cost, high-power Q-switched thulium-doped fibre laser based on NOLM-NALM hybrid configuration incorporating SWCNT polymer film as a SA. Our laser operates via a mechanism that enables stable Q-switched lasing with high output power without a physical isolator. Single pulse energy of 1.7 μJ and average power of 197 mW at a wavelength of 1885 nm have been achieved with the pump power of 1 W at 1550 nm. So far, this is the highest pulse energy/average power achieved in Q-switched TDF lasers employing SWCNT polymer composite based SA using only standard fibre components. The demonstrated laser system features a genuine all-fibre cavity structure. Future work with optimised laser cavity with SA for higher power output is expected. By the time the paper had been submitted and been undergoing the review process, we have noticed that similar cavity configuration in TDFL has been reported by Swiss group, however, for CW operation only^63^.

## Methods

### Preparation of Laser Ablation Single Wall Carbon Nanotube Polymer Composite

The fabrication procedure of our nanotube based polymer composite is similar to the previous reports^54^,^64^,^65^,^66^. The single wall carbon nanotube (SWCNT) is produced using a laser ablation technique. The reaction temperature in the laser ablation unit was 1372 °C and the reaction was run in high purity N_2_ gas. The targets consisted of 99.999% graphite with less than 10% wt. of a mixture of Ro, Rh and Mo mixed in. The laser was a Nd:YAG laser operating with a wavelength of 1064 nm, a pulse frequency of 10 Hz and pulse width of 20 ns. The reaction provides SWCNT with nominal mean diameter of 1.55 nm (+/−0.2 nm) as determined from optical absorption spectroscopy measurements^67^.

The raw SWCNTs (4 mg) were dispersed in (25 ml) DI water by (55 mg Sodium dodecylbenzenesulfonate) surfactant assisted sonication by using a NanoRuptor (Diagenode) processor for 1 h at 21 kHz and 250 W. The dispersion was then subjected to ultracentrifugation (Beckman Coulter Optima Max-XP, MLS 50 rotor) for 25 k r.p.m at 17 °C to remove large SWCNT bundles. Several grams of PVA powder was added to water and dissolved. Then both resulting SWNTs dispersions were mixed at a ratio of 3:1 with the PVA solution. The prepared suspension was poured into a petri dish and the water gradually evaporated over the period of one week. Eventually, the resulted dark optical transparent film formed was removed from the petri dish and hence a freestanding film was obtained.

### Characterisation of SWCNT PVA composite by optical absorption/transmission and Raman Spectroscopy

The pure PVA film and SWCNT-PVA composite were examined by absorption spectroscopy with commercially available Lambda1050 Perkin Elmer UV-NIR spectrometer equipped with PMT, InGaAs, and PbS 3-detector module for better sensitivity across the whole spectral range.

We used standard Micro Raman spectrometer (Renishaw 1000) equipped with DPSS laser (λ_ex_ = 532 nm) in our experiments. Raman system was calibrated using silica at 520 cm^1^. All Spectra were collected using 50× objective and 20 mW power using 10% filter with one accumulation of 20 seconds detector time. Measurements were carried out at room temperature. The excellent homogeneity of SWCNT composite with no visible SWCNT aggregates was confirmed by Raman microscope observation. Thus, we can have only a minor light scattering on SWCNT aggregates in our saturable absorption experiments.

### Nonlinear Saturable Absorption Measurement of SWCNT PVA SA

**Figure 6:**
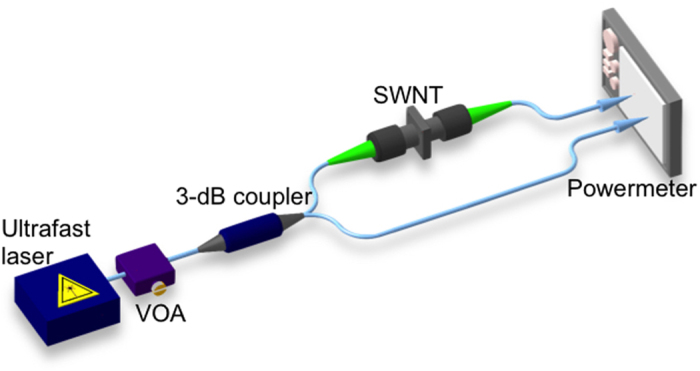
Schematic picture of the power-dependent characteristics measurements.

The nonlinear optical absorption measurement of the SA was characterised by using a self-made 500-fs thulium-doped passively mode-locked fibre laser, operating at 1940 nm, as probe light source. The laser setup and output pulse parameters are described in details in^61^. The SA was sandwiched between two fibre ferrules using a standard fibre mating sleeve. The probe light source was followed by a variable attenuator and then separated into two arms using a standard 50/50 fused coupler after connecting to an isolator in order to prevent any spurious reflection back to the laser cavity. One arm of the light source served as a reference, the other arm taking the measurement was connected to the SA. The power of the probe light source launched to the SA sample ranges from 0 to 3.5 mW. The absorption decrease of the SWCNT samples with the peak power increase was approximated according to the formula:


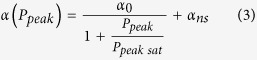


here α_0_ – modulation depth, α_ns_ – non-saturable absorption, P_peak sat_ – saturation peak power. The schematic is shown below in Fig. 6.

### Laser Components Specification

The Thulium-doped aluminum-silica (0.8 wt% thulium, 3.6 wt% aluminum) glass fiber (TDF), used in the fibre laser cavity as the gain medium, has a nominal group velocity dispersion (GVD) of β_2_ = −81 ps^2^/km at 1.9 μm. The active fibre features a 10 μm core diameter and 125 μm cladding diameter with λc ≈ 2.2 μm cut off wavelength. The non-saturated absorption at 1.56 μm pumping wavelength is 60 dB/m. The nominal absorption at the central operation wavelength ~1885 nm is 20.5 dB/km.

The ports of all passive components, i.e. couplers and WDM, and fibre pigtails constitute SMF-28 fibre only in out configuration. SMF-28 fibre has a GVD of β_2_ = −75 ps^2^ km^−1^ with estimated losses of 14.11 dB/km at 1900 nm. Because the TDF and SMF-28 fibres have similar mode field diameter, it allows a minimum splicing losses of <0.02 dB for the two splicing joints between SMF-28 and TDF. The total loss of all cavity components is estimated to be <1 dB.

### Measurement facilities for laser performance characterisation

The optical spectrum analyser we used in the experiment is from Yokogawa features a measurement range of 1700–2400 nm with a resolution of 0.05 nm. The photodiode used to measure the laser pulse is from EOT with a bandwidth of 12.5 GHz(ET-5000F). The oscilloscope is from Tektronics (TDS2012). The power meter used to characterise the output power is photodiode from Thorlabs (PM100D with S302C).

## Additional Information

**How to cite this article**: Chernysheva, M. *et al*. High Power Q-Switched Thulium Doped Fibre Laser using Carbon Nanotube Polymer Composite Saturable Absorber. *Sci. Rep.* **6**, 24220; doi: 10.1038/srep24220 (2016).
